# Evaluation of an Association of Blood Homocysteine Levels With Gastric Cancer Risk From 27 Case–Control Studies

**DOI:** 10.1097/MD.0000000000003700

**Published:** 2016-05-20

**Authors:** Wei Xu, Yuelei Cheng, Huirong Zhu

**Affiliations:** From the Department of Medical Oncology, Shuguang Hospital, Shanghai University of Traditional Chinese Medicine, Shanghai, China.

## Abstract

High blood homocysteine levels may risk gastric cancer. However, observational studies have been plagued by chance, bias, confounding, or reverse causality. In this study, we assessed the relationship between blood homocysteine levels and gastric cancer by using a Mendelian randomization method, which is independent of nongenetic confounding.

We took 2 steps to perform Mendelian randomization analysis. First, we evaluated the methylenetetrahydrofolate reductase (MTHFR) C677T association with gastric cancer by a meta-analysis of case-control studies including 7566 patients with gastric cancer and 10 640 control subjects from 27 Case–Control studies. Second, MTHFR C677T polymorphism, which affects the blood homocysteine levels, was used as an instrumental variable to calculate the risk and estimate the association of gastric cancer with this single nucleotide polymorphism (SNP). We obtained an estimate to the association of blood total homocysteine levels with this SNP from a meta-analysis of Genome-Wide Association Studies (GWAS), which comprises a total of 44 147 individuals.

In our Mendelian randomization analysis, we demonstrated a significant effect of the blood homocysteine levels on gastric cancer risk, representing an OR of 2.56 (95% CI = 2.41–2.72; *P* = 5.0×10^−4^) for gastric cancer per 1-SD increase in the natural log-transformed blood total homocysteine levels.

We proved that there is a causal relationship between blood total homocysteine and risk of gastric cancer, and this study will add insight into the treatment and pathology research of gastric cancer.

## Abstract 2

Supplemental Digital Content is available in the text

## INTRODUCTION

Gastric cancer (GC) becomes to be a commonly-seen health problem and the second deadliest in the world. In general, men's morbidity rate of GC is twice as high as women's in developing countries.^1^ In 2008, about 989 600 new GC patients and 738 000 deaths occurred in the world.^2,3^ From 2005 to 2009, the 5-year survival rate of GC patients is <40% in most of the countries except Japan and South Korea.^4^ Although we have controlled the decrease in the incidence of GC from the past cancer statistics, by changing diet structure and lifestyle, and reducing chronic *Helicobacter pylori* infection, the carcinogenesis of gastric undergoes multistage and multifactorial processes. Under similar environmental circumstances, the individuals with genetic factors are believed as crucial roles in the pathogenesis of GC would suffer from GC, whereas others do not, showing that there are complicated interactions between gene and gene or gene and environment.

The methylenetetrahydrofolate reductase (MTHFR) C677T polymorphism is a well-characterized genetic mutation. MTHFR plays a key reduction of the enzyme that carries out an irreversible catalyzing of 5, 10-methylene-tetrahydrofolate (5, 10-MTHF) to 5-methyl-tetrahydrofol-ate (5-MTHF) in the folate cycle, which recent researches have suggested the deficiency associated with a quickly growing number of cancers.^5,6^ C677T of the MTHFR as a consequence of gene hypomethylation results in amino acid product changing from alanine to valine,^7^ leading to reduction of enzyme activity and higher blood total homocysteine levels.^8^ A recent genome-wide association study of meta-analysis has confirmed the relationship of these variant genotypes with blood total homocysteine and reduction of enzyme activity.^9^ Cross-sectional and prospective studies and meta-analysis of 75 000 cases and 93 000 controls suggest that hyperhomocysteinemia be linked with increasing risk of GC, implicating that high Hcy levels might be the risk factor of GC.^10^ However, it is difficult to interpret these findings because of very large degrees of heterogeneity observes across studies such as confounding factors, reverse causation, selection bias, and so on.

A major limitation of observational studies is the difficulty in distinguishing between causal and spurious associations due to problems of confounding and reverse causation. Mendelian randomization (MR) is a study in which genetic variants are employed as instrumental variables to estimate the uncompounded effects of an exposure (in this study, Hcy) on a disease (in this study, GC). This approach based on the use of genes (in this study, MTHFR C677T) as instrumental variables has been proposed to assess causality and to provide estimates of the effect of modifiable intermediate phenotypes on disease unaffected by classical confounding or reverse causation, whenever randomized clinical trials are not feasible.^11^ This approach has provided new insights into the pathology of several diseases, such as myocardial infarction, type 2 diabetes, and all-cause (such as cancer and others disease but not cardiovascular) mortality.^12–15^ As the time of disease-onset is often poorly recognized clinically and MR studies do assess the effect of lifetime exposures, MR methods could be particular relevant towards understanding the etiology of GC.

In this study, a causal relationship between plasma total homocysteine and GC is established by conducting a Mendelian randomization analysis based on the MTHFR C677T polymorphism as an instrumental variable.

## METHODS

### Data on Gene Associations with GC Risk

To estimate the association of the MTHFR C677T polymorphism with GC risk, meta-analysis searching electronic databases was performed including PubMed and EMBASE, using the following search keywords.

(“stomach or gastric or cardia” and “cancer or neoplasm or carcinoma”), (“methylenetetrahydrofolate reductase” or “MTHFR”), and (“polymorphism,” “SNP,” or “genetic polymorphism”). The latest search was done in September 2015. We also screened the references from studies retrieved from AlzGene (http://www.alzgene.org) to collect all published genetic studies about GC. Reference lists of relevant articles were manually reviewed to look for additional studies. Studies included in the current meta-analysis had to meet the following criteria: (1) case-control, used to assess the relationship between MTHFR C677T polymorphism and GC risk, (2) sufficient genotype or allele data were presented to estimate the odds ratios (ORs) and 95% confidence intervals (95% CIs). When multiple studies were reported on the same or overlapping data, we chose the latest or largest population. Studies were excluded if there were (1) no detailed genotype frequency; (2) case reports, family-based studies, abstracts, editorials, and review articles. When multiple publications reported the same population, only the most recent one with the largest sample sets was selected for this meta-analysis. The 2 reviewers (Wei Xu, Yuelei Cheng) selected the articles independently according to the above criteria and then discussed the articles until they reached a consensus on each study used for the meta-analysis. The following data were independently extracted from each qualified article according to a fixed protocol: first author's name, publishing year, country and ethnicity of population, source of controls, the number of cases and controls, and the Hardy–Weinberg Equilibrium (HWE) in controls. If the information is not integrated, the authors of the publications would be contacted via E-mail for more detailed data. The methodological qualities of the included studies were accessed by 2 authors respectively according to the Newcastle Ottawa Scale (NOS) (http://www.ohri.ca/programs/clinical_epidemiology/oxford.asp). The NOS criteria consist of 3 aspects: selection, comparability, and exposure. Scores ranged from 0 stars (worst) to 9 stars (best) and a score ≥7 indicated that a study was of high quality. Dissent was settled as described above. This meta-analysis was based on previous published studies, and thus on ethical approval and patient consent were required.

### Data on Gene Associations With Plasma Total Homocysteine

The estimates of the effective sizes of the MTHFR C677T polymorphism on the plasma total homocysteine levels were based on the findings of a recent GWAS meta-analysis. The meta-analysis included data from a total of 44,147 Caucasian individuals of European ancestry derived from 10 GWAS on tHcy concentrations.^16^

### Statistical Analysis

Hardy–Weinberg equilibrium (HWE) of genotypes distribution in the control group was checked by the χ^2^-test and *P* < 0.05, which were considered as significant disequilibrium. The studies with controls not in HWE were subjected to a sensitivity analysis. The pooled odds ratios (ORs) with their 95% confidence intervals (95% CIs) were calculated to evaluate the strength of the association between MTHFR C677T polymorphism and GC risk based on different genetic models: allele model (T vs C), homozygous model (TT vs CC), heterozygous model (CT vs CC), dominant model (TT + CT vs CC), and recessive model (TT vs CT + CC). Statistical heterogeneity between eligible studies was evaluated using the Cochran's Q statistic and *I*^2^ test. *P* < 0.1 and *I*^2^ exceeding 50% indicated substantial heterogeneity across studies. A random-effects model was chosen to perform the meta-analysis. On the other hand, if the fixed-effects model was selected, subgroup analysis was conducted according to ethnicity (Asians, Caucasians, and Mixed) and study design. A power calculation was conducted using Power and Sample Size Calculation version 3.1.2 (http://biostat.mc.vanderbilt.edu/twiki/bin/view/Main/PowerSampleSize). Begg's funnel plot^17^ and Egger's regression test^18^ were used to search for publication bias and a *P* value > 0.05, suggesting that no significant publication bias had been detected. We calculated an MR estimate of the effect of the plasma total homocysteine levels on the risk of GC (OR _GC/hcy_) as log OR _GC/hcy_ = (log OR _GC/per__T-allele_)/ beta _hcy/per__T-allele_, as in previous studies. Log OR _GC/hcy_ is the (log) increase of GC risk by SD unit increase in the natural log-transformed plasma total homocysteine (MR estimate). Log OR _GC/per__T-allele_ is the (log) increase in GC risk per allele (gene-GC association). Beta _hcy/per__T-allele_ is the number of SD differences in the natural log-transformed plasma total homocysteine levels per allele (SD/allele) (gene-plasma total homocysteine association). The standard error (SE) of the MR estimate was derived from using the Delta method.^19,20^ The MR estimate was presented in terms of OR, by exponentiation the log OR _GC/hcy_. All *P* values were 2-sided. All above statistical analyses were performed using STATA software version 12.0 (STATA Corporation, College Station, TX).

## RESULTS

### Selection Process and Study Characteristics

The main characteristics of the included studies are summarized in Table 1 and the citation flow chart in Figure S1. Based on the search strategy, although there were 255 studies found, only 28 full-text articles were preliminarily identified for further detailed evaluation. According to the selection criteria, 1 study^21^ was excluded because their populations contain overlapping data.^22^ As a result, 27 relevant studies were included.^22–48^ Therefore, a total of 27 separate comparisons are enclosed.

**TABLE 1:**
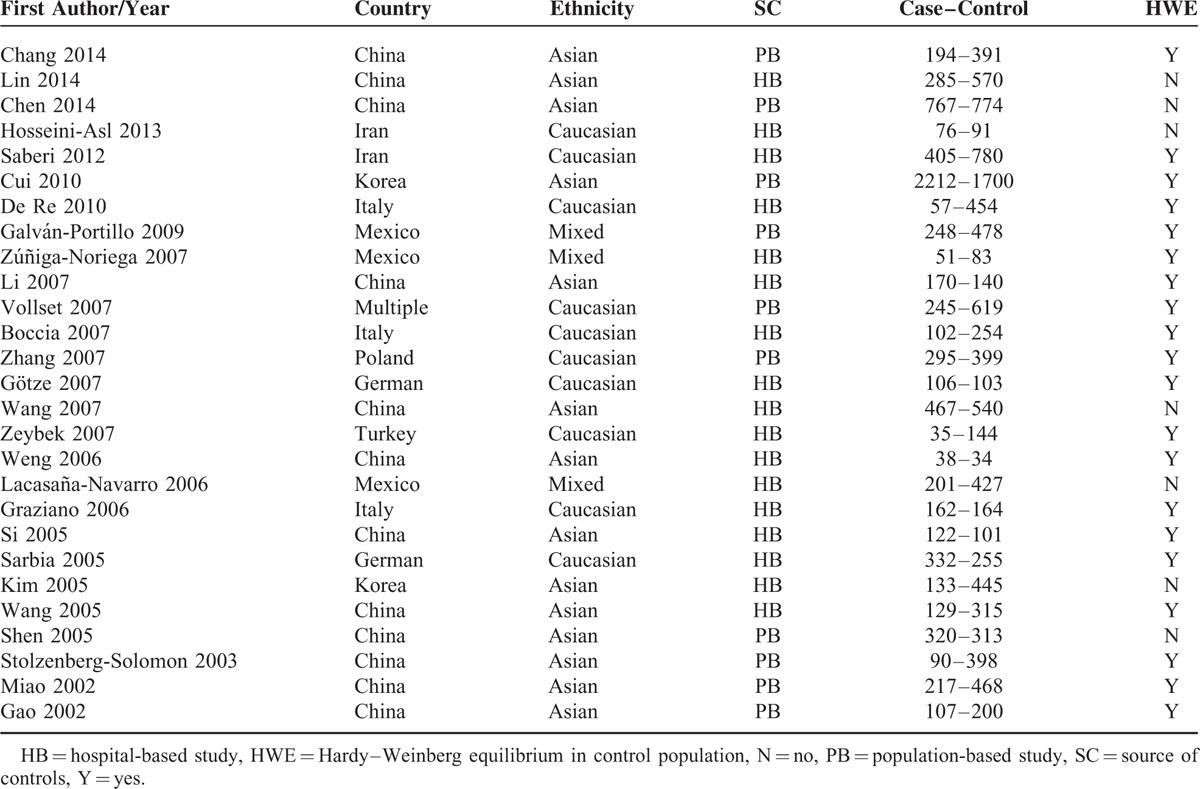
Characteristics of the Studies Included for the Gene-Homocysteine and Gene-GC Associations

### Association of MTHFR C677T Polymorphism With GC Risk

The main results of meta-analysis and heterogeneity test are summarized in Table 2 and Figure S2. The random-effects model was used to pool the result, if the results were not heterogeneous calculated by the fixed-effects model; else the random-effects model was used.^49,50^ The results of pooling all studies showed that the MTHFR C677T polymorphism was associated with increased GC risk in all genetic models (Figure S3). (T vs C: OR = 1.16, 95% CI = 1.06–1.27, *P* = 0.002; TT vs CC: OR = 1.36, 95% CI = 1.13–1.63,*P* = 0.001; CT vs CC: OR = 1.12, 95% CI = 0.97–1.29, *P* = 0.124; TT vs CT+CC: OR = 1.23, 95% CI = 1.09–1.40, *P* = 0.001; TT+CT vs CC: OR = 1.17, 95% CI = 1.02–1.35, *P* = 0.029).

**TABLE 2:**
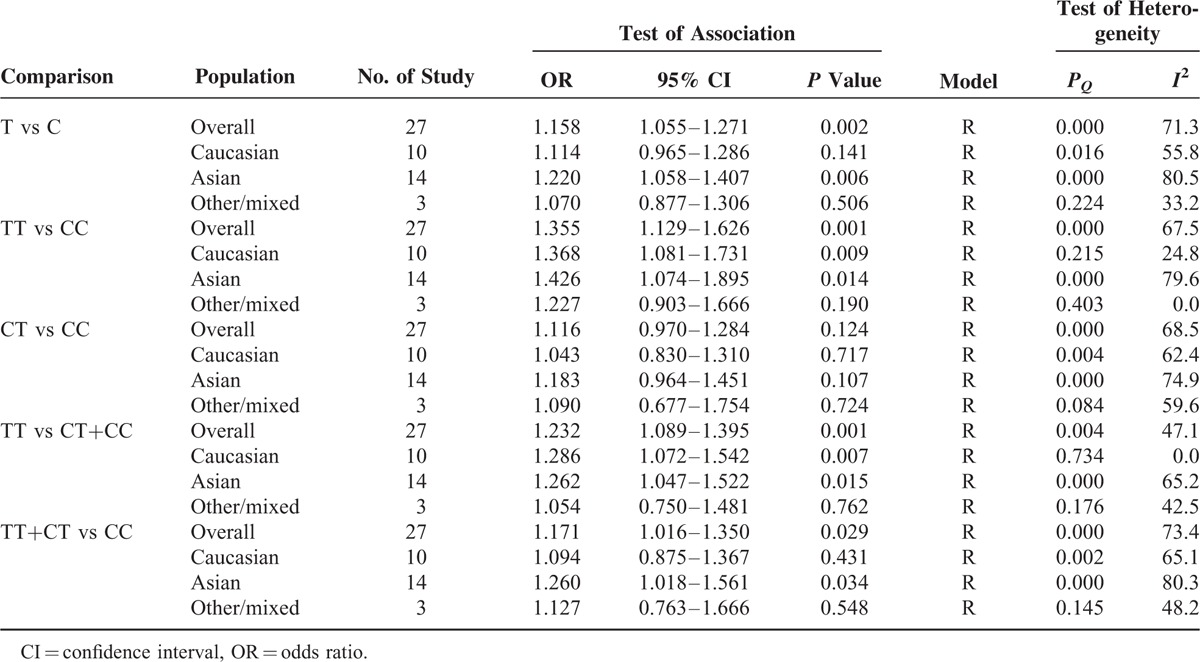
Meta-Analysis of the MTHFR C677T Polymorphism and Gastric Cancer

### Mendelian Randomization Analysis for the Association of Hcy With GC Risk

By combining 2 pooled estimates, OR _scz/per__T-allele_ from a meta-analysis of 27 case-control studies and beta _hcy/per__T-allele_ from a meta-analysis of genome-wide association studies,^16^ the meta-analysis of the MR estimate resulted in a statistically significant combined estimate of 2.56 (95% CI = 2.41–2.72; *P* = 5.0 × 10^−4^) representing the OR for GC per SD unit increase in hcy (see Figure S4). Considering that the null hypothesis value of unity was not covered by derived 95% CIs for predicted estimate, it was safe to the reject the null hypothesis of none causal relationship between plasma Hcy level and GC.

### Sensitivity Analysis and Publication Bias

The sensitivity analysis of individual studies by sequential removal and the control population—results were deviated from the HWE.^23,33,34,37,41,42,46^ All the significance of OR did not exclude the changes after these studies, and our results were robust and reliable. Potential bias was examined by Begg's funnel plot and Egg's test. The shape of the funnel plots in the picture did not show any obvious evidence of asymmetry. And also Egger's test provided no evidence for publication bias in the comparison of T allele vs C allele in the OR analysis in GC (*t* = 2.11, *P* = 0.045, see Figure S5 and Figure S6).

## DISCUSSION

In this study, using methylenetetrahydrofolate reductase C677T as instrumental variables, by the Mendelian randomization method, we demonstrated that a genetic increase in natural log-transformed plasma Hcy by 1 SD was associated with a 2.56-fold increased risk of GC. A recent meta-analysis of epidemiological studies (OR was 1.40, 95% CI [1.19–1.66]) also suggested a possible negative role of serum hcy levels on GC risk, but these findings are difficult to interpret due to the very large degree of heterogeneity across studies.^10^ Because of their observational nature, there is confounding and reverse causation limitation leading to the limitation of observational studies so that they can hardly provide conclusive evidence on the causality of an observed association. There are some potential confounding factors between hcy and GC, such as H. pylori infection, a risk factor for GC.^34,51^ Previous studies support that the folate plays an important role in raising the risk of GC^26,52^ and it can also lead to increasing levels of plasma homocysteine,^10^ so we cannot conclude that hcy levels increase risk of GC. However, if there is a link between a biomarker and a disease, the genes associated with the disease may also be associated with the disease.^53^ Based on the independent distribution law of genotypes, which were randomly assigned at meiosis unmodified by disease processes, Mendel explained the occurrence and development of the disease from the genetic level, avoiding the confounding factors and the reverse causality.

There is important clinical significance in identifying the relationship between Hcy levels and GC. Homocysteine is a key substance in the metabolism of sulfur amino acids from intermediate product of methionine cycle. The residue critical of methionine synthase (MTR) catalyzes the remethylation of homocysteine to methionine for maintaining the adequate intercellular folate level,^54^ which guides the DNA synthesis and methylation between homeostasis.^55^ Resulting therefrom hypomethylation associates with increased risk of gastric cancer. An elevated plasma level of Hcy (>14 μM) is termed as hyperhomocysteinemia (HHcy). The studies have shown that intake of folate above 310 μg/day may protect against the risk of gastric cancer.^52^ Folate consumption may interact with MTHFR C677T polymorphism to play a role in blood homocysteine levels with gastric cancer risk, just like previous studies of the effect of folate supplement on carcinogenesis.^27,31^ Epidemiological studies have found MTHFR c.677C > T homozygosity with lifelong associate hyperhomocysteinemia with increased risk of GC.^10^ Research studies show a relationship among low levels of folic acid, vitamin B12, high homocysteine levels, and GC. Increasing the levels of folic acid, vitamin B12 and B6 in the blood can reduce plasma homocysteine levels, further decreasing the risk of developing GC, which show that requiring dietary recommendations for GC prevention should depend on the individual genotype. This provides a new approach to the prevention and treatment of gastric cancer.

In the meta-analysis using MTHFR C677T as the instrumental variables, there is a strong relationship between gastric cancers and this SNP, and this finding shows the potential significance of gene C677T GC variation in MTHFR risk assessment in Asians and Caucasians.

In the present study, our data coming from GWAS meta-analysis for Hcy level (n = 44147)^16^ and current meta-analysis for GC risk (up to 6266 cases and 8250 controls)^56^ can provide a robust support for our findings, which suggests that the Mendelian randomization approach maybe be a potential and useful tool to assess the nature of the observed associations between putative risk factors and disease. However, there are some limitations to the present Mendelian randomization analysis. First, we only chose one of the genetic variants sites which the GWAS has provided several genetic variants, to some extent; and this approach may reduce the accuracy of our instrumental variables. Second, it seems impractical for us to exclude the pleiotropy of MTHFR C677T polymorphism as these data on other clinical parameters across C677T genotypes are rarely provided from most qualified literatures, requiring further confirmation. Third, it is the population stratification in our study which was composed of a mixed population and the source of Heterogeneity.

In conclusion, we found that there is a casual link between blood homocysteine levels and gastric cancer by the Mendelian randomization method, while increasing the level of folic acid reduces blood homocysteine levels, even decrease the risk of GC needing adequately powered randomized controlled trials. Our current findings offer new ideas for future clinical practice.

## Supplementary Material

Supplemental Digital Content
