# Supplementary material for: Evaluation of an Association of Blood Homocysteine Levels With Gastric Cancer Risk From 27 Case–Control Studies
Source: Medicine (Baltimore). 2016 May 20;95(20):e3700. doi: 10.1097/MD.0000000000003700 (PMC4902425; doi:10.1097/MD.0000000000003700)
Supplement: Supplemental Digital Content [file medi-95-e3700-s001.doc]

**Fig.S1:** A study flow diagram.

Screening

Included

Eligibility

Identification

Records identified through database searching
(n = 255)

Additional records identified through other sources
(n = 11)

Records after duplicates removed
(n = 115)

Records excluded
(n = 62)

26 articles excluded after full text review:

-Without original data (n = 12)

-No case-control (n = 10)

-No target outcomes (n = 1)

-Without details about sample and results (n =2)

-Overlap study (n = 1)

Studies included in this meta-analysis
(n = 27)

Records underwent title and abstract screening
(n = 115)

Full-text articles assessed for eligibility
(n = 53)

**Fig.S2:** The associated between MTHFR and GC risk in the random-effects model (T vs. C), stratiﬁed by study design. HB, hospital-based study; PB, population-based study.


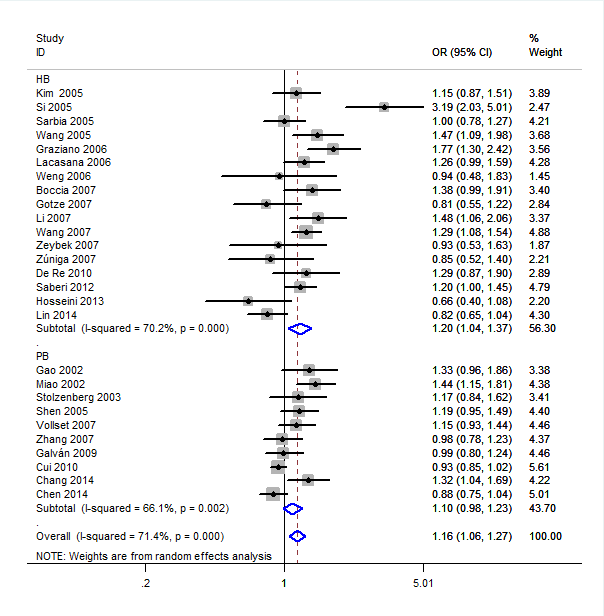


**Fig.S3:** The association between MTHFR and GC risk in the random-effects model (T vs. C)


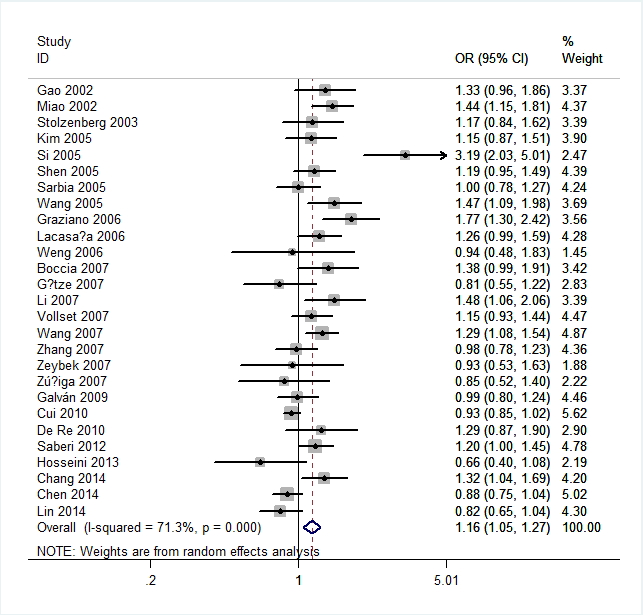


**Fig.S4:** Graphical representation of the Mendelian randomization approach. The risk assessment of gene-GC for T allele from our meta- analysis which included 7,566 cases and 10,640 controls, gene and plasma homocysteine risk assessment was based on the findings of a recent Genome-Wide Association Studies (GWAS) meta-analysis; through those two results we can calculate our Mendel randomization results. It represents a unit of homocysteine can increase the risk of GC by 2.56 times.


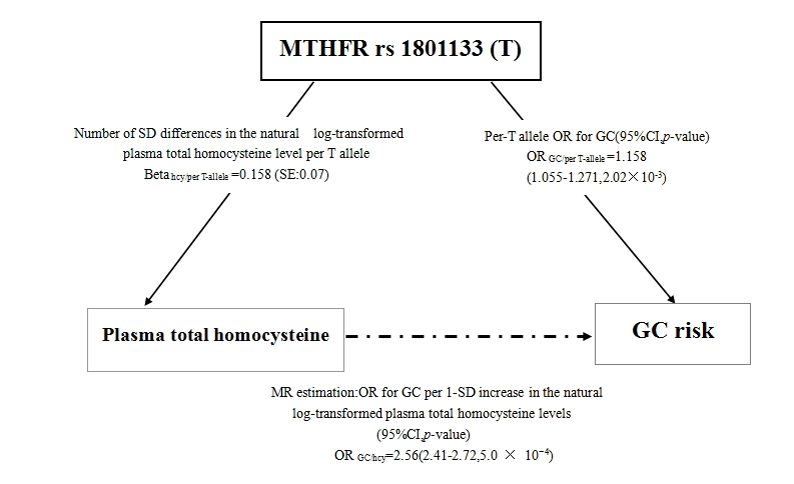


**Fig.S5** Funnel plot

**Fig.S6**
